# Extremely drug-resistant NDM-9-producing ST147 *Klebsiella pneumoniae* causing infections in Italy, May 2020

**DOI:** 10.2807/1560-7917.ES.2020.25.48.2001779

**Published:** 2020-12-03

**Authors:** Marco Falcone, Cesira Giordano, Simona Barnini, Giusy Tiseo, Alessandro Leonildi, Paolo Malacarne, Francesco Menichetti, Alessandra Carattoli

**Affiliations:** 1 Infectious Diseases Unit, Department of Clinical and Experimental Medicine, University of Pisa, Pisa, Italy; 2 The authors contributed equally this article; 3 Microbiology Unit, Azienda Ospedaliera Universitaria Pisana, Pisa, Italy; 4 Department of Anaesthesia and Critical Care Medicine, Azienda Ospedaliera Universitaria Pisana, Pisa, Italy; 5 Department of Molecular Medicine, Sapienza University of Rome, Rome, Italy

**Keywords:** Outbreak, NDM, Tuscany, carbapenem resistance, Colistin resistance, Tigecycline resistance, Fosfomycin resistance

## Abstract

A large outbreak of New Delhi metallo-beta-lactamase (NDM)-1-producing *Klebsiella pneumoniae* sequence type (ST) 147 occurred in Tuscany, Italy in 2018–2019. In 2020, ST147 NDM-9-producing *K. pneumoniae* were detected at the University Hospital of Pisa, Tuscany, in two critically ill patients; one developed bacteraemia. Genomic and phylogenetic analyses suggest relatedness of 2018–2019 and 2020 strains, with a change from NDM-1 to NDM-9 in the latter and evolution by colistin, tigecycline and fosfomycin resistance acquisition.

A large outbreak sustained by New Delhi metallo-beta-lactamase (NDM)-1-producing *Klebsiella pneumoniae* sequence type (ST) 147 occurred in Tuscany, Italy in 2018–2019 [1,2]. In 2020, NDM-9-producing *K. pneumoniae* were isolated in the same geographical area. In this study, comparisons at genomic level of these recent isolates with strains representative of the 2018–2019 outbreak were carried out. The 2020 strains were ST147 and had acquired resistance to colistin, tigecycline, and fosfomycin.

## Case reports

In May 2020, two critically ill patients were respectively colonised or infected by extensively drug-resistant (XDR), NDM-9-producing *K. pneumoniae* at the University Hospital of Pisa, Tuscany, Italy.

The first patient (P1) was admitted to the intensive care unit (ICU) of the hospital for a minor surgical intervention related to a cerebral trauma 1 year earlier. At admission, a rectal swab culture was positive for NDM-producing *K. pneumoniae* (strain Kp-P1; identification by MALDI-ToF MS, Bruker Daltonics, Bremen, Germany). Susceptibility to a series of antibiotics was assessed (Merlin ITGN Panel, Tecan automated system, Tecan Trading AG, Männedorf, Switzerland), using minimum inhibitory concentrations (MICs) breakpoints established by the European Committee on Antimicrobial Susceptibility Testing (EUCAST) [3]. The isolated strain resulted resistant to all tested antibiotics, including colistin, fosfomycin (agar-dilution method; AD Fosfomycin, Liofilchem, Roseto degli Abruzzi, Italy) and tigecycline (Table 1). It was susceptible to cefiderocol (SensiTitre Cefiderocol MIC panel CMP1SHIH, Thermo Fisher, Waltham, MA, USA). No significant infections were diagnosed in this patient during the hospital stay.

**Table 1:** Minimum inhibitory concentrations of ST147 NDM-producing *Klebsiella pneumoniae* strains described in this study, Pisa, Italy (n = 8 strains)

Antibiotic	*Klebsiella pneumoniae* strains/isolates Minimum inhibitory concentrations in mg/L							
	Kp-P1 Kp-P2	Kp-8Pi	Kp-9Pi	Kp-11Pi	Kp-12Pi	Kp-14Pi	Kp-16Pi	Kp-17Pi
Ceftazidime	> 128	> 128	> 128	> 128	> 128	> 128	> 128	> 128
Cefepime	> 32	32	> 32	> 32	> 32	> 32	> 32	> 32
Ceftriaxone	> 4	> 4	> 4	> 4	> 4	> 4	> 4	> 4
Cefiderocol	2	NT	NT	NT	NT	NT	NT	NT
Meropenem	> 8	8	8	8	8	4	8	64
Imipenem	> 8	4	8	8	8	4	16	> 16
Ciprofloxacin	> 2	> 2	> 2	> 2	> 2	> 2	> 2	> 2
Levofloxacin	> 4	> 4	> 4	> 4	> 4	> 4	> 4	> 4
Amikacin	> 16	> 16	> 16	> 16	> 16	> 16	> 16	> 16
Gentamycin	> 4	> 4	> 4	> 4	> 4	> 4	> 4	> 4
SXT	> 4	> 4	> 4	> 4	> 4	> 4	> 4	> 4
Tigecycline	2	0.25	0.25	0.5	0.5	0.25	1	0.25
Fosfomycin	> 128	≤ 16	≤ 16	≤ 16	≤ 16	≤ 16	32	16
Colistin	16	< 0.5	< 0.5	< 0.5	16	<0.5	16	< 0.5
Aztreonam	> 32	> 32	> 32	> 32	> 32	> 32	> 32	> 32
Ceftazidime/avibactam	> 8/4	> 8/4	> 8/4	> 8/4	> 8/4	> 8/4	> 8/4	> 8/4
Ceftazidime/avibactam plus aztreonam^a^	FICI 0.03	Synergistic	NT	NT	NT	NT	NT	NT

ATM: aztreonam; AVI: avibactam; FICI: fractional inhibitory concentration index; MIC: minimum inhibitory concentration; NDM: New Delhi metallo-beta-lactamase; NT: not tested; SXT: sulfamethoxazole-trimethoprim.

MICs were obtained using the Merlin ITGN Panel, Tecan automated system, Tecan Trading AG, Switzerland.

^a^ FICI ATM MIC = 0.25 mg/L with AVI at concentration of 4 mg/L.

A second patient (P2) was admitted 5 days later to the same ICU because of a traumatic injury. The patient had no history of previous hospitalisation. Throat and rectum swabs taken at admission yielded cultures negative for carbapenem-resistant Gram-negative bacilli. A rectal swab taken on day 7 post admission, however, led to the detection of an NDM-producing *K. pneumoniae* by PCR for the *bla*_NDM_ gene. On day 13, the patient developed a central venous catheter-related bacteraemia due to NDM-producing *K. pneumoniae* (strain Kp-P2). The susceptibility profile of strain Kp-P2 was identical to that of strain Kp-P1 (Table 1). Checkerboards were set up with twofold dilutions of aztreonam (ATM; 0.03 to 128 mg/L) and ceftazidime-avibactam (CAZ-AVI; 1–0.25 to 64–16 mg/L) as previously described [4]: the combination resulted fully synergistic (fractional inhibitory concentration index, FICI = 0.03; Table 1) against strain Kp-P2. The patient was treated with intravenous CAZ-AVI 2.5 g every 8 hours plus ATM 2 g every 8 hours. The clinical conditions rapidly improved but the patient had a second episode of bloodstream infection (BSI) by NDM-producing *K. pneumoniae* on day 56. A second course of CAZ-AVI plus ATM was initiated, the patient’s condition improved leading to discharge from ICU after 69 days. No relapse of infection was observed after 30 days from discharge.

## Ethical statement

The study was conducted according to the principles stated in the Declaration of Helsinki. The local Ethics Committee (Comitato Etico Area Vasta Nord Ovest) approved the study (IRB n 61185). Study participants signed the informed consent.

## Genomic analysis of the 2020-NDM-9 ST147 *Klebsiella pneumoniae* strains

Genomic DNAs were purified from Kp-P1 and Kp-P2 *K. pneumoniae* strains (Macherey Nagel DNA extraction kit, Düren, Germany) to build up paired-end libraries (Nextera XT), which were sequenced using the Illumina MiSeq instrument with 2x300PE protocol (Illumina Inc, San Diego, California, United States). De novo assembly of reads was performed by SPAdes v3.8 (https://w3.iss.it/site/aries/). Kp-P1 and Kp-P2 genomes were released under Bioproject PRJNA667843.

Kp-P1 and Kp-P2 strains were assigned to ST147 by in silico multilocus sequence typing (MLST; https://bigsdb.pasteur.fr/klebsiella/klebsiella.html), the same ST as the NDM-1-producing *K. pneumoniae* clone that caused a large outbreak in Tuscany in 2018–2019 [1,2].

A phylogenetic tree using parsimony was built based on single nucleotide polymorphisms (SNP) (kSNP v3.0; https://w3.iss.it/site/aries/) in Kp-P1 and Kp-P2 genomes, as well as in 21 ST147 genomes of strains isolated in Pisa during the 2018–2019 outbreak and in 13 genomes of unrelated, internationally identified ST147 strains that were downloaded from GenBank.

ST147 sequences of recent and prior isolates in Pisa formed a cluster, which was distinct from epidemiologically unrelated ST147 sequences. Within the Pisa cluster, Kp-P1 and Kp-P2 formed a further group with a subset of strains (Kp-11Pi, Kp-12Pi and Kp-16Pi) isolated in 2019. The sequences in this sub-cluster were highly related (Figure).

**Figure:**
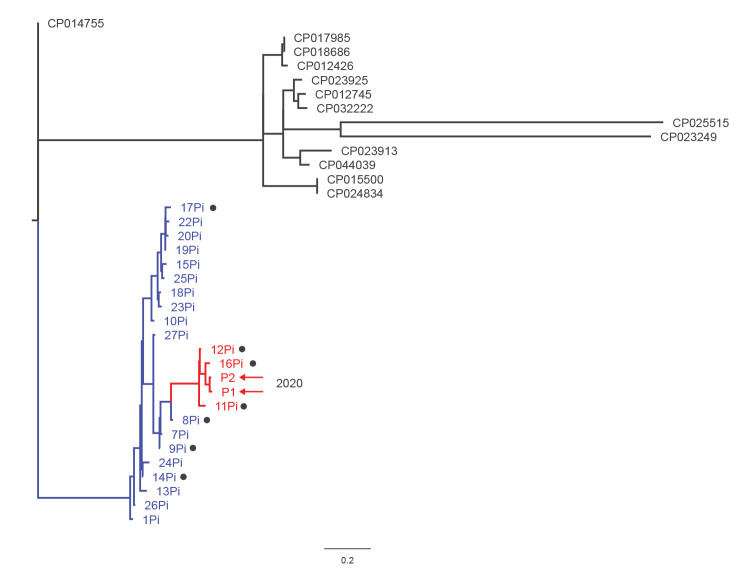
Phylogenetic tree to analyse ST147 *Klebsiella pneumoniae* sequences from isolates of two patients in Pisa, Italy, 2020 (n = 36 sequences)

CDS: coding sequence; SNP: single nucleotide polymorphism; ST: sequence type.

All sequences analysed in the phylogenetic analysis are of ST147. The parsimony SNP analysis was performed by the kSNP3 (Galaxy version 3.1) software at the ARIES public Galaxy server (https://w3.iss.it/site/aries/). The phylogenetic tree was visualised using the Fig Tree programme v. 1.4.3 (http://tree.bio.ed.ac.uk/software/figtree/). The phylogenetic tree was based on the sequences of Kp-P1 and Kp-P2 genomes (highlighted by red arrows) as well as those of 34 genomes, including 21 from strains isolated during the 2018–2019 outbreak in Pisa (in blue or red font) and 13 from epidemiologically-unrelated strains (in black). The sequences of the 13 unrelated strains were downloaded from GenBank (accession numbers are indicated on the tree leaves). Black dots indicate the seven genomes selected for protein alignment analysis (https://rast.nmpdr.org/seedviewer.cgi). Translations of the CDSs in these genomes were aligned to those of Kp-P1 and Kp-P2 genome CDSs for comparison. The scale bar indicates the extent of genetic change by estimating the average number of nucleotide substitutions per site.

## Evolution of *Klebsiella pneumoniae* ST147 antimicrobial resistance

The Kp-P1 and Kp-P2 strains carried the *bla*_NDM-9_ gene, which presented one amino-acid substitution (E152K) with respect to *bla*_NDM-1_ gene identified in 2018–2019 isolates. A complex array of plasmids was identified in Kp-P1 and Kp-P2 strains, including derivatives of the pKpQIL, pKPN3, pNDM-MAR and IncR plasmids (PlasmidFinder v2.1 [5]). It was not possible to obtain complete assembly of plasmids for these strains. No relevant differences were found in acquired resistance genes (Table 2¸ ResFinder v3.2 [6]) and replicon content, in particular comparing the two Kp-P1 and Kp-P2 strains with the three closely related Kp-11Pi, Kp-12Pi and Kp-16Pi strains. This observation suggested that the observed phenotypical variations in MICs for colistin (16 mg/L for Kp-1, Kp-2, Kp-12Pi and Kp-16Pi vs < 0.5 mg/L for the other five strains), fosfomycin (> 128 mg/L for Kp-1 and -2 vs ≤ 32 mg/L for the remaining strains) and tigecycline (2 mg/L for Kp-1 and -2 vs ≤ 1 mg/L for the other strains) were likely due to acquired chromosomal mutations rather than to horizontal transfer of additional plasmid-mediated resistance genes.

**Table 2:** Genetic features of 2020-ST147 *Klebsiella pneumoniae* strains described in this study compared with six strains representative of the 2018–2019-ST147 outbreak, Pisa, Italy (n = 8 strains)

Strain SRA	Year	Acquired beta-lactamases	Other acquired resistance genes	Replicon content	TygR, ColR, and FosR chromosomal loci
Kp-P1 SRR12785639	2020	NDM-9, CTX-M-15, OXA-1, OXA-9, TEM-1A	*armA, aadA1, aac(6')-Ib, aph(3')-VI, aph(3')-1A, catB3, dfrA5, mph(E), msr(E), qnrS1, sul1, sul2*	HI1B-FIB(pNDM-Mar); FIIk-FIB(pKpQIL); FIB(pKpN3); R; FIB(pKPHS1); Col(pHAD28); ColRNAI	*ramR* (C^364^→T)-ΔRamR *mgrB* (A^7^→T)-ΔMgrB *glpT* (C^401^→T)-ΔGlpT
Kp-P2 SRR12785638	2020	NDM-9, CTX-M-15, OXA-1, OXA-9, TEM-1A	*armA, aadA1, aac(6')-Ib3, aph(3')-VI, aph(3')-1A, catB3, dfrA5, mph(E), msr(E), qnrS1, sul1, sul2*	HI1B-FIB(pNDM-Mar); FIIk-FIB(pKpQIL); FIB(pKpN3); R; N; FIB(pKPHS1); Col(pHAD28); ColRNAI	*ramR* (C^364^→T)-ΔRamR *mgrB* (A^7^→T)-ΔMgrB *glpT* (C^401^→T)-ΔGlpT
Kp-8Pi SRR12518027	2018	NDM-1, CTX-M-15, OXA-1, OXA-9, TEM-1A	*armA, aadA1, aac(6')-Ib, aph(3')-VI, aph(3')-1A, catB3, dfrA5, mph(E), msr(E), qnrS1, sul1, sul2*	HI1B-FIB(pNDM-Mar); FIB(pKpQIL); R; FIB(pKPHS1)	RamR wt MgrB wt GlpT wt
Kp-9Pi SRR12518023	2018	NDM-1, CTX-M-15, OXA-1, OXA-9, TEM-1A	*armA, aadA1, aac(6')-Ib, aph(3')-VI, aph(3')-1A, catB3, dfrA5, mph(E), msr(E), qnrS1, sul1, sul2*	HI1B-FIB(pNDM-Mar); FIB(pKpQIL); R; FIB(pKPHS1)	RamR wt MgrB wt GlpT wt
Kp-11Pi SRR12518041	2018	NDM-1, CTX-M-15, OXA-1, OXA-9, TEM-1A	*armA, aadA1, aac(6')-Ib, aph(3')-VI, aph(3')-1A, catB3, dfrA5, mph(E), msr(E), qnrS1, sul1, sul2*	HI1B-FIB(pNDM-Mar); FIB(pKpQIL); FIIk-FIB(pKpN3); R; FIB(pKPHS1)	*ramR* (C^364^→T)-ΔRamR MgrB wt GlpT wt
Kp-12Pi SRR12518021	2019	NDM-1, CTX-M-15, OXA-1, OXA-9, TEM-1A	*armA, aadA1, aac(6')-Ib, aph(3')-VI, aph(3')-1A, catB3, dfrA5, mph(E), msr(E), qnrS1, sul1, sul2*	HI1B-FIB(pNDM-Mar); FIB(pKpQIL); FIIk-FIB(pKpN3); R; FIB(pKPHS1); ColRNAI	*ramR* (C^364^→T)-ΔRamR PmrA mut MgrB wt GlpT wt
Kp-14Pi SRR12518062	2019	NDM-1, CTX-M-15, OXA-1, OXA-9, TEM-1A	*armA, aadA1, aac(6')-Ib, aph(3')-VI, aph(3')-1A, catB3, dfrA5, mph(E), msr(E), qnrS1, sul1, sul2*	HI1B-FIB(pNDM-Mar); FIB(pKpQIL); R; FIB(pKPHS1); ColRNAI	RamR wt MgrB wt GlpT wt
Kp-16Pi SRR12518060	2019	NDM-1, CTX-M-15, OXA-1, OXA-9, TEM-1A	*armA, aadA1, aac(6')-Ib, aph(3')-VI, aph(3')-1A, catB3, dfrA5, mph(E), msr(E), qnrS1, sul1, sul2*	HI1B-FIB(pNDM-Mar); FIB(pKpQIL); FIIk-FIB(pKpN3); R; FIB(pKPHS1); ColRNAI	*ramR* (C^364^→T)-ΔRamR *mgrB* (A^7^→T)-ΔMgrB *glpT* (G^1088^→C)-GlpT mut
Kp-17Pi SRR12518059	2019	NDM-1, CTX-M-15, OXA-1, OXA-9, TEM-1B	*armA, aadA1, aac(6')-Ib, aph(3')-VI, aph(3')-1A, catB3, dfrA5, mph(E), msr(E), qnrS1, sul1, sul2*	HI1B-FIB(pNDM-Mar); FIB(pKpQIL); R; FIB(pKPHS1); X4	RamR wt MgrB wt GlpT wt

GlpT: glycerol-3-phosphate transporter; mut: mutation; NDM: New Delhi metallo-beta-lactamase; OXA: oxacillinase; PmrA: multi-drug resistance efflux pump PmrA; RamR: resistance antibiotic multiple (Ram)A regulator; SRA: Sequence Read Archive accession number at the National Center for Biotechnology Information database (NCBI; https://www.ncbi.nlm.nih.gov/); ST: sequence type; wt: wild type.

Kp-P1 and Kp-P2 are the *K. pneumoniae* strains derived from patient 1 and patient 2, in 2020 in Pisa, who are described in this study. The rest of the strains in the table are representative of the 2018–2019 outbreak in Pisa.

Kp Δ is used when mutations caused premature termination of translation of the deduced protein sequence. Mut indicates that the deduced protein sequence is mutated with respect to the wt amino-acid sequence.

Protein basic local alignment search tool (BLASTP) analysis was performed on deduced coding sequences (CDSs) obtained by translation of Kp-P1 and Kp-P2 genomes (https://rast.nmpdr.org/seedviewer.cgi) in comparison with CDSs deduced from seven genomes, with different susceptibility to colistin, fosfomycin and tigecycline antibiotics (Table 1), which included also those of the three Kp-11Pi, Kp-12Pi and Kp-16Pi strains.

A mutation in the *ramR* gene (C^364^→T), creating a premature stop codon at position 122 was identified in Kp-P1, Kp-P2, Kp-11Pi, Kp-12Pi, and Kp-16Pi strains, respectively. The resistance antibiotic multiple A regulator (RamR) protein is otherwise 194 amino acids in the wild type (GenBank accession number: ARJ36702.1). RamR has been reported to act as the negative regulator of resistance antibiotic multiple A (RamA) that is the positive regulator of the AcrAB efflux pump system. Depletion of RamR results in constitutive activation of the efflux pump increasing MICs for tigecycline [7]. In this study, depletion of RamR increased tigecycline MICs from 0.25 mg/L (observed in strains with a wild type RamR) to 0.5 mg/L in strains Kp-11Pi and Kp-12Pi, to 1 mg/L in strain KP-16Pi and to 2 mg/L in Kp-P1 and Kp-P2 strains (Table 1). MIC differences observed among *ramR* mutants were likely due to additional mutations in the Kp-P1 and Kp-P2 genomes that were not identified in this study.

A mutation (A^7^→T) in the *mgrB* gene created a premature stop codon and the complete depletion of the MgrB protein (GenBank accession number: QDC86160.1), the regulator of *phoP* gene expression, in colistin-resistant Kp-P1, Kp-P2 and Kp-16Pi strains, respectively (Tables 1,2) [8]. A substitution G53V was identified in the PmrA protein (reference GenBank nr. KDM30728) of the other colistin resistant Kp-12Pi strain (Table 1). No *mcr* genes were detected in the genomes under investigation.

Fosfomycin resistance in Kp-P1 and Kp-P2 was due to a mutation in the *glpT* gene creating a premature stop codon at position 134 (wild type glycerol-3-phosphate transporter (GlpT) is 448 amino acids; GenBank accession number: KLY15715) causing the depletion of the GlpT (Table 2). Defects in one of two GlpT or uptake hexose phosphate (UhpT) transporters lead to reduced fosfomycin uptake into the bacterial cells causing increasing MICs for this antibiotic [9,10]. A substitution S79L was also found in the hexose-phosphate uptake two-component transcriptional response regulator UhpA protein in Kp-P1 and -2 strains. No mutations were found in MurA, UhpT, FosA, CyaA, Crp, PtsI, UhpB and UhpC proteins related to fosfomycin importation and metabolism [11-13].

## Discussion

A total of 1,645 cases with NDM-carbapenem-resistant Enterobacteriaceae (CRE)-positive microbiological samples were identified in the period from 1 November 2018 to 31 October 2019 in Tuscany [1]. In the same period, at the University Hospital of Pisa 705 patients were colonised or infected by CRE, and among them 388 were reported as testing positive for NDM-CRE (data not shown). The outbreak in Tuscany was dominated by the *K. pneumoniae* ST147 clone (1,495 isolates; 90.9%), characterised by the presence of the *bla*_NDM-1_ gene [1,2]. Data from the Pisa University Hospital showed that 98% of NDM-producing strains were *K. pneumoniae.* The outbreak was controlled but some sporadic cases occurred in Tuscany later in 2019 [1]. The outbreak clone was resistant to almost all beta-lactam antibiotics, including expanded-spectrum cephalosporins, carbapenems, beta-lactamase inhibitor combinations, aztreonam, and aminoglycosides. Susceptibility to colistin and fosfomycin was detected in most of the isolates collected during the outbreak. The combination CAZ-AVI/ATM resulted synergistic in vitro and associated with clinical benefit [2,14].

In this study, we describe the resistance genetic features of XDR ST147 *K. pneumoniae* strains isolated in 2020. These isolates were genetically related to the 2018–2019 outbreak clone, but had the NDM-9 instead of the NDM-1 carbapenemase variant, with no apparent changes in beta-lactam susceptibility. Mutations in chromosomally encoded genes conferred resistance to tigecycline, fosfomycin and colistin. From a clinical perspective, the 2020 strains represent a new variant, which may become a notable challenge for clinicians, because of the extended resistant profile.

The new variant likely emerged from a subgroup of highly related ST147 strains from the 2018–2019 outbreak. These strains harboured mutations in RamR and MgrB proteins, implicated in tigecycline and colistin resistance, respectively. In particular, the RamR depletion detected in three of the strains, coincided, in one of them, with the MgrB depletion, similar what is observed for the new variant. RamR followed by MgrB protein depletions may have been intermediate steps in the evolution of the variant. The acquired fosfomycin resistance appears to be the most novel characteristic of the emerging ST147 variant and appeared associated with a defect in the GlpT. Few *K. pneumoniae* strains have been reported with this fosfomycin resistance mechanism [13], therefore it represents an interesting case of resistance evolution. Further investigation is needed to understand in which setting and by which combination therapy this XDR clone has been selected.

The persistence of *K. pneumoniae* ST147 isolation suggests that this clone can be considered as endemic in the Pisa University Hospital and there is risk of spread to other hospitals in Tuscany and elsewhere. Several actions, such as systematic screening of hospitalised patients, contact precautions, and cohorting of colonised/infected patients are ongoing to contain the spread of this clone.

## Data availability

Kp-P1 and Kp-P2 genomes have been released under Bioproject PRJNA667843
